# Response Predictors of Repetitive Neuromuscular Magnetic Stimulation in the Preventive Treatment of Episodic Migraine

**DOI:** 10.3389/fneur.2022.919623

**Published:** 2022-07-28

**Authors:** Corinna Börner, Tabea Renner, Florian Trepte-Freisleder, Giada Urban, Paul Schandelmaier, Magdalena Lang, Matthias F. Lechner, Helene Koenig, Birgit Klose, Lucia Albers, Sandro M. Krieg, Thomas Baum, Florian Heinen, Mirjam N. Landgraf, Nico Sollmann, Michaela V. Bonfert

**Affiliations:** ^1^Division of Pediatric Neurology and Developmental Medicine and LMU Center for Children With Medical Complexity, Dr. von Hauner Children's Hospital, LMU Hospital, Ludwig-Maximilians-Universität, Munich, Germany; ^2^Department of Diagnostic and Interventional Neuroradiology, School of Medicine, Klinikum rechts der Isar, Technical University of Munich, Munich, Germany; ^3^TUM-Neuroimaging Center, Klinikum rechts der Isar, Technical University of Munich, Munich, Germany; ^4^Department of Neurosurgery, School of Medicine, Klinikum rechts der Isar, Technical University of Munich, Munich, Germany; ^5^Department of Diagnostic and Interventional Radiology, University Hospital Ulm, Ulm, Germany

**Keywords:** headache, migraine, neurostimulation, non-invasive neuromodulation, repetitive peripheral magnetic stimulation, myofascial trigger point, preventive migraine therapy, migraine prevention

## Abstract

**Background:**

Repetitive neuromuscular magnetic stimulation (rNMS) of the trapezius muscles showed beneficial effects in preventing episodic migraine. However, clinical characteristics that predict a favorable response to rNMS are unknown. The objective of this analysis is to identify such predictors.

**Methods:**

Thirty participants with a diagnosis of episodic migraine (mean age: 24.8 ± 4.0 years, 29 females), who were prospectively enrolled in two non-sham-controlled studies evaluating the effects of rNMS were analyzed. In these studies, the interventional stimulation of the bilateral trapezius muscles was applied in six sessions and distributed over two consecutive weeks. Baseline and follow-up assessments included the continuous documentation of a headache calendar over 30 days before and after the stimulation period, the Migraine Disability Assessment Score (MIDAS) questionnaire (before stimulation and 90 days after stimulation), and measurements of pain pressure thresholds (PPTs) above the trapezius muscles by algometry (before and after each stimulation session). Participants were classified as responders based on a ≥25% reduction in the variable of interest (headache frequency, headache intensity, days with analgesic intake, MIDAS score, left-sided PPTs, right-sided PPTs). *Post-hoc* univariate and multivariate binary logistic regression analyses were performed.

**Results:**

Lower headache frequency (*P* = 0.016) and intensity at baseline (*P* = 0.015) and a migraine diagnosis without a concurrent tension-type headache component (*P* = 0.011) were significantly related to a ≥25% reduction in headache frequency. Higher headache frequency (*P* = 0.052) and intensity at baseline (*P* = 0.014) were significantly associated with a ≥25% reduction in monthly days with analgesic intake. Lower right-sided PPTs at baseline were significantly related to a ≥25% increase in right-sided PPTs (*P* = 0.015) and left-sided PPTs (*P* =0.030). Performance of rNMS with higher stimulation intensities was significantly associated with a ≥25% reduction in headache intensity (*P* = 0.046).

**Conclusions:**

Clinical headache characteristics at baseline, the level of muscular hyperalgesia, and stimulation intensity may inform about how well an individual patient responds to rNMS. These factors may allow an early identification of patients that would most likely benefit from rNMS.

## Introduction

Migraine is one of the most prevalent neurological disorders worldwide, with more than one billion affected people in 2016 and a significant impact on health-related quality of life, work productivity, and social relationships (1–3). Migraine is countervailed by a multimodal approach of lifestyle management, psychoeducation, psychotherapeutic intervention, and pharmacotherapy (4–6). Medication for migraine attacks is well-established and widely used; yet, responsiveness to prophylactic treatment varies and treatment adherence is often poor (e.g., due to side effects or insufficient adjustment of dosage) (7, 8). Against this background, innovative non-pharmacological treatment options are highly required (4, 8–10).

Neurostimulation represents a non-pharmacological treatment alternative that has emerged over the recent years (11–14). It aims at modifying the complex processes and interactions in and in-between the central, peripheral, and/or autonomous nervous system through externally applied electrical or magnetically induced stimuli. Several approaches exist, including: transcranial magnetic stimulation (TMS), transcranial direct current stimulation (tDCS), occipital nerve stimulation (ONS), transcutaneous supraorbital nerve stimulation (tSNS), transcutaneous vagus nerve stimulation (tVNS), and remote electrical neurostimulation (REN) of cutaneous sensory afferents of the upper arm (10, 15–22).

Furthermore, repetitive neuromuscular magnetic stimulation (rNMS) has been introduced lately, targeting to the neck and shoulder muscles to prevent attacks in episodic migraine (23–25). Specifically, rNMS has been reported to be safe, feasible, well-tolerated, and well-accepted (23–25). Moreover, promising effects of rNMS in terms of a reduction in headache frequency, headache intensity, migraine-associated disability, and muscular hypersensitivity have been reported (23–25). It is hypothesized that rNMS intervenes at the terminal branches of the motor and afferent nerves in the region within the induced electromagnetic field, thus directly and indirectly leading to an increase of proprioceptive sensation (26, 27). The trigemino-cervical complex (TCC) serves as a gateway for this bottom-up approach and its translation to modulate the central mechanisms of nociception (10, 28, 29).

However, rNMS demands a commitment in terms of patient's and therapist's resources. Stimulators are increasingly available on the markets, but, they are still by far more expensive than devices for transcutaneous electrical nerve stimulation (tENS), which limits availability. Therefore, recommending rNMS for migraine prevention anticipates thorough consideration of which patient may benefit the most in the context of an individualized multimodal treatment paradigm. However, no data on the predictors of a treatment response to rNMS nor to any other neurostimulating approach are available. The aim of this study was to assess clinical headache and muscular characteristics as well as technical aspects of the stimulation protocol that are associated with a positive treatment response to rNMS regarding headache frequency, headache intensity, burden of migraine, frequency of analgesic intake, and level of muscular hyperalgesia.

## Methods

### Ethics

The protocols of the two non-sham-controlled studies that form the basis of the present analyses were approved by the institutional review boards of both universities of Munich. The studies were conducted in accordance with the Declaration of Helsinki. Written informed consent was obtained from all participants.

### Study Design

For the analysis of response predictors, the baseline, treatment, and follow-up charts of 30 participants who received rNMS to the upper trapezius muscles during two previous prospective non-sham-controlled clinical studies have been reviewed (23–25). The following inclusion criteria were applied during those studies: (1) age between 18 and 35 years, (2) episodic migraine [according to the German version of the headache questionnaire modified according to the International Classification of Headache Disorders (ICHD), 3rd beta edition (30–32)], (3) at least one active myofascial trigger point (mTrP) in one of the upper trapezius muscles (identified by a physiotherapist specialized in manual palpation of mTrPs), and (4) no metallic implants (e.g., pacemaker, cochlear implants). The following criteria were defined as exclusion criteria: (1) chronic migraine (≥15 headache days per month for >3 months) (30), (2) any neurological disorder except for primary headache, (3) intake of any medication for migraine prophylaxis, and (4) pregnancy.

During the previous studies, each participant underwent six sessions of rNMS in regular intervals during two consecutive weeks (e.g., Monday/Wednesday/Friday or Tuesday/Thursday/Saturday) (23–25). A Nexstim eXimia NBS System with a figure-of-eight stimulation coil was used for rNMS (version 4.3; Nexstim Oy, Helsinki, Finland). Before starting the first rNMS session, the stimulation intensity (% of maximum stimulator output) was determined individually for the trapezius muscles and was kept for both sides for the following sessions. Individual stimulation intensities were set by increasing the intensity in 5% steps until participants reported a discomfortable sensation (defined as a score of 5 on a 0–10 visual analog scale). Next, this intensity was decreased by 5% so that a comfortable and non-painful stimulation over 15 min was possible (23–25). Stimulation targeted to the left and right upper trapezius muscles – focusing on the mTrP with the highest intensity of referred pain—for 15 min per side during each session. Stimulation of each side consisted of 20 bursts with a total of 6.000 stimuli and a 20-Hz frequency. A single burst lasted 15 s and was composed of 300 stimuli, followed by a relaxation time of 30 s.

### Baseline and Follow-up Assessments

The German version of the headache questionnaire modified according to the ICHD (3rd beta edition) (30–32), the headache calendar of the German Migraine and Headache Society (DMKG) (33), and the Migraine Disability Assessment Score (MIDAS) questionnaire (34, 35) were applied. The presence of aura symptoms and an association with tension-type headache (TTH) were documented as well.

To evaluate the headache frequency and characteristics, participants were asked to fill in the headache calendar of the DMKG on a daily basis in the 30 days before the first rNMS session. Numerous items of each headache attack like date, trigger mechanisms (stress, relaxation, disturbance of sleep-awake rhythm, or menstruation), intensity, duration, quality, localization, forerunning symptoms (scintillating scotoma, paresthesia, or aphasia), concomitant symptoms (nausea, vomiting, photophobia, phonophobia or odor-sensitivity), drug intake, dosage form, and pain relief were recorded with the help of the calendar. Subsequently, the participants filled in the headache diary during the course of the 30 days after the last rNMS intervention, defined as the follow-up period.

Moreover, participants were advised to fill in the MIDAS questionnaire to evaluate the impairment by headache events in different aspects of daily life before and after the application of rNMS. As the MIDAS questionnaire is evaluating a period of 90 days, this questionnaire had to be completed prior to the first rNMS session (evaluating the 90 pre-interventional days) and 90 days after the last session (evaluating the 90 post-interventional days). Measurements of PPTs were performed with an analog algometer by applying pressure with its rubber tip of 1 cm^2^ perpendicularly to the determined mTrPs. The pressure was increased with a velocity of 1 kg/s/cm^2^ until the local PPT was attained according to the participant. This algometry was conducted three times per side before and after rNMS during each of the six sessions, and the average of each three measurements was calculated afterwards (36, 37). In this context, the PPT was defined as the cut-off value between mere pressure and pressure-induced painful perception (37–41).

### Statistical Analysis

SPSS (version 25.0; IBM SPSS Statistics for Windows, Armonk, NY, USA) was used for statistical data analyses. Response to rNMS was investigated by categorizing participants into responders and non-responders according to a ≥25% response criterion for the following outcomes: (1) headache frequency, (2) headache intensity, (3) MIDAS score, (4) days with analgesic intake per month, (5) left-sided PPT, and (6) right-sided PPT. The following potential predictors were evaluated: (1) age, (2) headache type (episodic migraine, episodic migraine with concurrent TTH diagnosis), (3) pre-interventional headache frequency, (4) pre-interventional headache intensity, (5) pre-interventional MIDAS score, (6) pre-interventional days with analgesic intake per month, (7), pre-interventional left-sided PPT, (8) pre-interventional right-sided PPT, and (9) stimulation intensity. Differences between pre- and post-interventional values of predictor variables were assessed using paired *t*-tests.

We performed univariate binary logistic regression analyses to assess the influence of each potential predictor on the outcome variable. To assess whether the regression model is better fitted than a null model, the Omnibus test was used. Further, multivariate binary logistic regression analyses (with a backward elimination approach) were used to evaluate the combined influence of potential predictors on the response (≥25% response rate) to rNMS. The Benjamini-Hochberg procedure with a false discovery rate (FDR) of 10% was used to adjust for multiple testing. The statistical significance for all tests was set at α = 0.05.

## Results

Thirty participants who received rNMS applied to the bilateral trapezius muscles were included in the analysis. All participants had a diagnosis of episodic migraine, and 10 subjects had an additional diagnosis of concurrent TTH (33.3%). Participants were on average 24.8 ± 4.0 years old (age range: 19–35 years) and 29 of the participants were female (96.7%). Demographics as well as baseline and follow-up characteristics are summarized in Table 1.

**Table 1:** Descriptive statistics of *n* = 30 patients affected by episodic migraine participating in two pilot studies of repetitive neuromuscular magnetic stimulation applied to the trapezius muscles.

	**Descriptive Statistics**					**Paired** ***t*****-Test**	
	***N***	**%**	**Mean**	**SD**	**Range**	**T**	***P*-value**
**Age (years)**			24.80	3.96	19–35		
**Gender**							
Female	29	96.70					
Male	1	3.30					
**Headache type**							
Migraine without TTH	20	66.70					
Migraine with TTH	10	33.30					
**Headache frequency (days/month)**							
Pre-interventional			8.17	4.50	2–26	2.88	0.007^a^
Post-interventional			6.33	4.38	1–20		
**Headache intensity (10-point VAS scale)**							
Pre-interventional			5.23	1.37	2.52–7.5	−0.73	0.473
Post-interventional			5.40	1.33	3.00–7.43		
**MIDAS score**							
Pre-interventional			26.33	13.89	2–58	6.24	<0.001^a^
Post-interventional			15.27	12.30	1–47		
**Analgesic intake (days/month)**							
Pre-interventional			3.63	2.58	0–8	1.25	0.233
Post-interventional			3.10	2.44	0–9		
**PPT left (kg)**							
Pre-interventional			2.02	0.89	0.97–4.17	−4.26	<0.001^a^
Post-interventional			2.68	1.16	0.80–5.33		
**PPT right (kg)**							
Pre-interventional			2.19	0.83	0.87–3.83	−4.10	<0.001^a^
Post-interventional			2.94	1.15	0.63–5.04		
rNMS intensity (% of maximum stimulator output)			24.23	4.66	15–35		

a *Results significant at α = 0.05. TTH, tension-type headache; VAS, visual analog scale; MIDAS, migraine disability assessment; PPT, pressure pain threshold; rNMS, repetitive neuromuscular magnetic stimulation*.

Response set to a level of ≥25% was achieved in 50% of this cohort (*n* = 15) in terms of decreases in headache frequency, in 13% of participants (*n* = 4) in terms of decreases in headache intensity, in 73% of participants (*n* = 22) in terms of decreases in the MIDAS score, in 50% of participants (*n* = 15) in terms of decreases in monthly days with analgesic intake, and in 53% of participants (*n*= 16) in terms of increases in left-sided PPTs as well as in 60% of participants (*n* = 18) in terms of increases in right-sided PPTs, respectively. The results of the univariate analyses are summarized in Table 2.

**Table 2:** Results of the univariate analyses of response predictors of repetitive neuromuscular magnetic stimulation applied to the trapezius muscles in patients affected by episodic migraine.

**Predictor**	**Omnibus Test**		**B**	**SE**	***P*-value**	**Exp (B)**	**95% CI [Exp (B)]**
	**Chi^2^**	***P*-value**					
**25% responder rate of headache intensity**							
rNMS intensity	6.27	0.012	0.37	0.18	0.046^a^	1.44	1.01–2.07
**25% responder rate of MIDAS score**							
Headache frequency	4.02	0.045	−0.21	0.13	0.110	0.81	0.63–1.05
Days with analgesic intake/month	3.93	0.048	−0.35	0.19	0.067	0.71	0.49–1.02
**25% responder rate of days with analgesic intake**							
Headache intensity	4.42	0.035	0.62	0.32	0.053	1.86	0.99–3.48
Days with analgesic intake/month	6.79	0.009	0.42	0.18	0.019^a^	1.52	1.07–2.15
**25% responder rate of left-sided PPT**							
Right-sided PPT	6.00	0.014	−1.25	0.58	0.030^a^	0.29	0.09–0.89
**25% responder rate of right-sided PPT**							
rNMS intensity	4.75	0.029	0.20	0.10	0.050^a^	1.22	1.00–1.49

a *Results significant at α = 0.05 and after adjusting for multiple testing using the Benjamini-Hochberg correction with 10% FDR. Only predictors for which the Omnibus test was significant are displayed in this table. B, unstandardized beta (regression coefficient); SE, standard error of the unstandardized beta; Exp(B), expected beta; CI, confidence interval of the expected beta; MIDAS, Migraine Disability Assessment; PPT, pressure pain threshold; rNMS, repetitive neuromuscular magnetic stimulation; FDR, false discovery rate*.

The multivariate analyses revealed the following results (Table 3): headache type as well as pre-interventional headache frequency and headache intensity were significantly associated with responsiveness in terms of a ≥25% reduction in headache frequency. Responders had on average lower pre-interventional headache frequency (responders: 7.4 ± 3.4 days/month; non-responders: 8.9 ± 5.4 days/month) and intensity (responders: 5.0 ± 1.3 points; non-responders: 5.5 ± 1.5 points on a 10-point visual analog scale). In addition, responders were more often diagnosed with migraine without a concurrent diagnosis of TTH [responders: 12 participants without a TTH diagnosis (80%); non-responders: 8 participants without a TTH diagnosis (53.3%)]. The pre-interventional headache intensity was significantly associated with responsiveness in terms of a ≥25% reduction in monthly days with analgesic intake. The association of pre-interventional headache frequency with responsiveness showed a statistical trend (*P* = 0.052). Responders had on average higher pre-interventional headache frequency (responders: 9.0 ± 5.6 days/month; non-responders: 7.3 ± 3.0 days/month) and intensity (responders: 5.7 ± 1.4 points; non-responders: 4.7 ± 1.1 points on a 10-point visual analog scale).

**Table 3:** Results of the multivariate analyses of response predictors of repetitive neuromuscular magnetic stimulation applied to the trapezius muscles in *n* = 30 patients affected by episodic migraine.

**Predictor**	**B**	**SE**	***P*-value**	**Exp(B)**	**95% CI [Exp (B)]**
**25% responder rate of headache frequency**					
Headache type	4.13	1.63	0.011^a^	–	–
Headache frequency	−0.41	0.17	0.016^a^	0.66	0.47–0.93
Headache intensity	−1.41	0.58	0.015^a^	0.25	0.08–0.76
**25% responder rate of headache intensity**					
rNMS intensity	0.37	0.18	0.046^a^	1.44	1.01–2.07
**25% responder rate of days with analgesic intake**					
Headache intensity	1.00	0.40	0.014^a^	2.71	1.23–5.99
Headache frequency	0.23	0.12	0.052	1.26	1.00-1.60
**25% responder rate of left-sided PPT**					
Right-sided PPT	−1.25	0.58	0.030^a^	0.29	0.09–0.89
**25% responder rate of right-sided PPT**					
Left-sided PPT	2.02	1.07	0.060	7.51	0.92–61.37
Right-sided PPT	−2.83	1.16	0.015^a^	0.06	0.01–0.58
rNMS intensity	0.25	0.14	0.076	1.28	0.97–1.69

a *Results significant at α = 0.05 and after adjusting for multiple testing using the Benjamini-Hochberg correction with 10% FDR. All variables not mentioned in the table were excluded in the prior steps of regression analysis. B, unstandardized beta (regression coefficient); SE, standard error of the unstandardized beta; Exp(B), expected beta; CI, confidence interval of the expected beta; PPT, pressure pain threshold; rNMS, repetitive neuromuscular magnetic stimulation; FDR, false discovery rate*.

The pre-interventional right-sided PPT was significantly associated with responsiveness in terms of a ≥25% increase in the right-sided PPTs. Responders had on average lower pre-interventional right-sided PPTs (responders: 2.0 ± 0.9; non-responders: 2.4 ± 0.7). The pre-interventional right-sided PPT was significantly associated with responsiveness in terms of a ≥25% increase in the left-sided PPTs. Responders had on average lower pre-interventional right-sided PPTs (responders: 1.9 ± 0.7; non-responders: 2.6 ± 0.8). Stimulation intensity was significantly associated with responsiveness in terms of a ≥25% reduction in headache intensity. Responders received rNMS with higher stimulation intensities on average (responders: 29.3 ± 4.4% of maximum stimulator output; non-responders: 23.5 ± 4.3% of maximum stimulator output).

No statistically significant predictors were identified for responsiveness in terms of a ≥25% reduction in MIDAS scores (*P* > 0.05).

## Discussion

In migraine research, neurostimulation methods are emerging non-invasive, non-pharmacological approaches, for which efficacy data is available but information on clinical baseline characteristics associated with positive treatment response are still lacking (11, 12). This study points at clinical headache and muscular characteristics as well as a technical factor as potential predictors for a beneficial response to rNMS in participants with episodic migraine. Reductions in headache frequency (from 8.17 to 6.33 headache days per month) and in MIDAS scores (from 26.3 reflecting severe disability to 15.3 reflecting moderate disability) were observed after application of rNMS compared to the baseline status, whereas no significant changes were found for headache intensity or duration.

Participants achieving a reduction in headache frequency of at least 25% had on average lower headache frequency, lower headache intensity, and were more often diagnosed with migraine without a TTH component at baseline. Responsiveness in terms of ≥25% reduction in monthly analgesic intake days was associated with higher mean headache intensity at baseline and higher headache frequency by trend. Regarding muscular involvement, participants achieving a ≥25% increase in right- and left-sided PPTs had on average lower baseline right-sided PPTs. From the technical perspective, participants with a decrease of at least 25% in headache intensity received rNMS with higher mean stimulation intensities. All those findings are in agreement with the current concept of migraine pathophysiology, which includes not only central pain mechanisms but also points at muscular involvement of the neck muscles (7, 42–44). Clinically, particularly the involvement of the upper trapezius muscles has been described more pronounced in migraine than in episodic TTH (45, 46). Supported by muscular imaging by advanced techniques like muscle T2 mapping of the trapezius muscles, the clinical signs might be considered surrogates of muscular neuroinflammation (47, 48). This imaging finding could be seen in line with the framework of the TCC (28, 29).

The level of sensitization and impairment of the nociceptive feedback control systems may eventually be more easily amenable by a tailored treatment approach the lower the baseline headache frequency and intensity are. This may imply to consider a neuromodulatory approach early during the course of disease, before perpetuation of the disorder. With respect to this assumption, a follow-up rNMS study involving patients suffering from chronic migraine would be of interest, as well as long-term follow-up investigations to assess the sustainability of the beneficial effects in different subgroups of patients (e.g., episodic migraine vs. high-frequent episodic migraine vs. chronic migraine). Treating migraine *via* the bottom-up approach allows the modulation of the afferent input to the TCC and, in consequence, of the central pain processing mechanisms (10, 28, 29). Since TTH is associated with different pathophysiological mechanisms, patients with migraine having a concurrent TTH component might respond to rNMS to a lesser extent (49–52).

Patients who are more frequently or more intensely affected by migraine may also use more medication for pain relief. Hence, a decrease of the intake of analgesics is likely to reflect a lower headache frequency and/or intensity as a positive response to rNMS. The better treatment response in patients with a higher level of muscular hyperalgesia supports the concept of the bottom-up approach, as well. In this regard, rNMS targeting the part of the trapezius muscles that is included in the TCC is particularly effective in patients with a high level of muscular involvement. Specifically, the impact of the stimulation intensity on the outcome might reflect a dose-effect relationship. Given the novelty of the rNMS approach, no comparisons of different stimulation protocols have been conducted yet.

Of note, we chose a reduction of ≥25% as responder rate since clinical experience support that responder rates lower than 50% are also clinically meaningful in the context of non-pharmacological preventive treatments (53). This is especially true for the cohort of this study since it involves participants suffering from frequent episodic migraine (up to 26 headache days per month).

Data on the predictors of treatment response to other non-invasive methods of neurostimulation (e.g., TMS, tDCS, or tENS of cranial nerves) for the prevention of migraine is lacking so far. Only one study examined potential predictors for the response to invasive ONS in refractory chronic headache (54). It showed that shorter unilateral headache attacks and prior response to a pharmacologically induced occipital nerve block were associated with a greater likelihood for a positive response to invasive ONS (54).

When interpreting the results of this analysis the following limitations should be respected. First, the sample size is low, which does not allow for an extrapolation to the general population of migraine patients. Second, the analysis relies on data retrieved from not-sham-controlled pilot studies, which is why placebo effects in the context of response level cannot be excluded. Further research is needed to evaluate the association of clinical as well as muscular characteristics and technical aspects to treatment response for neurostimulation therapy. In addition, future studies should investigate rNMS in a higher number of patients as well as in sham-controlled settings to assess and correct for a potential placebo effect. Future studies could for instance assess further predictors like age at onset of migraine, overall duration of migraine (55), number of local spots of muscular hyperalgesia (i.e., mTrPs), fluid biomarkers (e.g., calcitonin gene-related peptide), or biomarkers based on novel muscular imaging methods (e.g., T2 mapping derived from magnetic resonance imaging of the trapezius muscles) (47, 48). Since our results derive from a cohort of young adults with episodic migraine, future studies should include other migraine cohorts as well (e.g., pediatric populations). Further, this study did not assess variables reflecting central sensitization (e.g., allodynia), and it did not systematically assess common comorbidities like depression or anxiety. Future studies should implement such comorbidities in their study design. In addition, different classifications of responsiveness should be considered, for example ≥25% vs. ≥50% response, excellent responders (56), full-length responders, or wearing-off responders (57). Moreover, the establishment of standardized protocols for treatment and for data collection during baseline and follow-up are necessary for reliable data analysis and bias exclusion (55, 58). The identification of potential predictors for the different neurostimulation approaches and for a larger cohort of patients could enable an individually tailored, efficacy-predicting tool (score chart) in a multimodal therapy setting (59).

## Conclusion

This analysis informs about predictors of treatment response to rNMS applied to the upper trapezius muscle in a cohort of young adults affected by episodic migraine. Findings demonstrate that some clinical headache characteristics at baseline (headache frequency, headache intensity, and headache diagnosis), the level of muscular hyperalgesia expressed by PPTs at baseline, as well as technical aspects during rNMS (stimulation intensity) may deliver information on how well an individual patient may respond to rNMS. These factors may allow early identification of patients who would experience benefits of rNMS based on their initial clinical presentation. This is important as rNMS represents an innovative and promising treatment approach that is, however, restricted to single headache centers at the current stage, only. Further, to establish a treatment option like rNMS in a cost- and time-efficient manner, the individual counseling on the treatment options in the context of a multimodal regimen should be based on all evidence available.

## Data Availability Statement

The raw data supporting the conclusions of this article will be made available by the authors, without undue reservation.

## Ethics Statement

The studies involving human participants were reviewed and approved by Ethikkommission TUM & LMU. The patients/participants provided their written informed consent to participate in this study.

## Author Contributions

TR and FTF conducted the clinical studies on which the analysis of the paper is based on with support of BK and HK and under the supervision of NS, FH, and MNL. CB and NS conducted the statistical analysis with support of TB and LA. CB, TR, MB, NS, SK, MNL, and FH discussed and interpreted the findings and gave their expert opinion with regards to all analyses. CB, TR, and MB drafted the manuscript. GU, PS, and ML compiled tables. All authors reviewed, commented on and contributed to the final manuscript. All authors have agreed to this final version of the article being submitted.

## Funding

MNL received grants from the Deutsches Zentrum für Luft- und Raumfahrt (DLR-PT). MNL and FH received a grant Innovationsfonds of the joint federal committee of health insurance companies (GBA) for a nation-wide study on an early multimodal intervention program for children with migraine. The work of MB is supported by the Bavarian Gender Equality Grant of the Free State of Bavaria, the German Migraine and Headache Society (DMKG), Germany, and the ZNS-Hannelore-Kohl Stiftung, Germany. The work of NS is supported by the Dr. Ing. Leonhard Lorenz Foundation, the German Migraine and Headache Society (DMKG), and the Joachim Herz Foundation.

## Conflict of Interest

The Division of Pediatric Neurology and Developmental Medicine, Dr. von Hauner Children's Hospital, LMU Hospital, Munich Germany is provided by an emFieldPro magnetic stimulator by Zimmer MedizinSysteme GmbH (Neu-Ulm, Germany). NS and SK received honoraria from Nexstim Plc (Helsinki, Finland). SK received honoraria from Brainlab AG (Munich, Germany), Spineart (Frankfurt, Germany), Ulrich Medical (Ulm, Germany), Zeiss Meditec (Jena, Germany), and Medtronic (Dublin, Ireland). The remaining authors declare that the research was conducted in the absence of any commercial or financial relationships that could be construed as a potential conflict of interest.

## Publisher's Note

All claims expressed in this article are solely those of the authors and do not necessarily represent those of their affiliated organizations, or those of the publisher, the editors and the reviewers. Any product that may be evaluated in this article, or claim that may be made by its manufacturer, is not guaranteed or endorsed by the publisher.

## Abbreviations

DMKG: German Migraine and Headache Society
ICHD: international classification of headache disorders
MIDAS: migraine disability assessment
mTrP: myofascial trigger point
ONS: occipital nerve stimulation
PPT: pressure pain threshold
QoL: quality of life
REN: remote electrical neurostimulation
rNMS: repetitive neuromuscular magnetic stimulation
TCC: trigemino-cervical complex
tDCS: transcranial direct current stimulation
tENS: transcutaneous electrical nerve stimulation
TMS: transcranial magnetic stimulation
TTH: tension-type headache
tSNS: transcutaneous supraorbital nerve stimulation
tVNS: transcutaneous vagus nerve stimulation.

## References

[1] Martelletti P Schwedt TJ Lanteri-Minet M Quintana R Carboni V Diener HC. My migraine voice survey: a global study of disease burden among individuals with migraine for whom preventive treatments have failed. J Headache Pain. (2018) 19:115. 10.1186/s10194-018-0946-z 30482181 PMC6755592

[2] Steiner TJ Stovner LJ Vos T Jensen R Katsarava Z. Migraine is first cause of disability in under 50s: will health politicians now take notice? J Headache Pain. (2018) 19:17. 10.1186/s10194-018-0846-2 29468450 PMC5821623

[3] Vo P Fang J Bilitou A Laflamme AK Gupta S. Patients' perspective on the burden of migraine in Europe: a cross-sectional analysis of survey data in France, Germany, Italy, Spain, and the United Kingdom. J Headache Pain. (2018) 19:82. 10.1186/s10194-018-0907-6 30203163 PMC6131678

[4] Bonfert M Straube A Schroeder AS Reilich P Ebinger F Heinen F. Primary headache in children and adolescents: update on pharmacotherapy of migraine and tension-type headache. Neuropediatrics. (2013) 44:3–19. 10.1055/s-0032-1330856 23303551

[5] Diener H-C Gaul C Kropp P. Therapie der Migräneattacke und Prophylaxe der Migräne, S1-Leitlinie. In: Deutsche Gesellschaft für Neurologie (Hrsg), Leitlinien für Diagnostik und Therapie in der Neurologie. (2018).

[6] Bonfert MV. Primäre Kopfschmerzen bei Kindern und Jugendlichen - Update 2019. Pädiatrie Up2date. (2019) 14:71–85. 10.1055/s-0043-115285

[7] Charles A. The pathophysiology of migraine: implications for clinical management. Lancet Neurol. (2018) 17:174–82. 10.1016/S1474-4422(17)30435-0 29229375

[8] Diener H-C Charles A Goadsby PJ Holle D. New therapeutic approaches for the prevention and treatment of migraine. Lancet Neurol. (2015) 14:1010–22. 10.1016/S1474-4422(15)00198-2 26376968

[9] Brighina F Raieli V Messina LM Santangelo G Puma D Drago F. Non-invasive brain stimulation in pediatric migraine: a perspective from evidence in adult migraine. Front Neurol. (2019) 10:364. 10.3389/fneur.2019.00364 31031695 PMC6473052

[10] Borner C Urban G Beaulieu LD Sollmann N Krieg SM Straube A. The bottom-up approach: non-invasive peripheral neurostimulation methods to treat migraine: a scoping review from the child neurologist's perspective. Eur J Paediatr Neurol. (2021) 32:16–28. 10.1016/j.ejpn.2021.02.008 33743386

[11] Schoenen J Roberta B Magis D Coppola G. Non-invasive neurostimulation methods for migraine therapy: the available evidence. Cephalalgia. (2016) 36:1170–80. 10.1177/0333102416636022 27026674

[12] Reuter U McClure C Liebler E Pozo-Rosich P. Non-invasive neuromodulation for migraine and cluster headache: a systematic review of clinical trials. J Neurol Neurosurg Psychiatry. (2019) 90:796–804. 10.1136/jnnp-2018-320113 30824632 PMC6585264

[13] Vukovic Cvetkovic V Jensen RH. Neurostimulation for the treatment of chronic migraine and cluster headache. Acta Neurol Scand. (2019) 139:4–17. 10.1111/ane.13034 30291633

[14] Moisset X Pereira B Ciampi de Andrade D Fontaine D Lanteri-Minet M Mawet J. Neuromodulation techniques for acute and preventive migraine treatment: a systematic review and meta-analysis of randomized controlled trials. J Headache Pain. (2020) 21:142. 10.1186/s10194-020-01204-4 33302882 PMC7726868

[15] Rapoport AM Bonner JH Lin T Harris D Gruper Y Ironi A. Remote electrical neuromodulation (REN) in the acute treatment of migraine: a comparison with usual care and acute migraine medications. J Headache Pain. (2019) 20:83. 10.1186/s10194-019-1033-9 31331265 PMC6734294

[16] Rapoport AM Lin T Tepper SJ. Remote electrical neuromodulation (REN) for the acute treatment of migraine. Headache. (2020) 60:229–34. 10.1111/head.13669 31913517

[17] Yarnitsky D Dodick DW Grosberg BM Burstein R Ironi A Harris D. Remote Electrical neuromodulation (REN) relieves acute migraine: a randomized, double-blind, placebo-controlled, multicenter trial. Headache. (2019) 59:1240–52. 10.1111/head.13551 31074005 PMC6767146

[18] Rodrigo D Acin P Bermejo P. Occipital nerve stimulation for refractory chronic migraine: results of a long-term prospective study. Pain Physician. (2017) 20:E151–9. 10.36076/2017.1.E151 28072807

[19] de Coo IF Marin JC Silberstein SD Friedman DI Gaul C McClure CK. Differential efficacy of non-invasive vagus nerve stimulation for the acute treatment of episodic and chronic cluster headache: a meta-analysis. Cephalalgia. (2019) 39:967–77. 10.1177/0333102419856607 31246132 PMC6637721

[20] Ordas CM Cuadrado ML Pareja JA de-Las-Casas-Camara G Gomez-Vicente L Torres-Gaona G. Transcutaneous supraorbital stimulation as a preventive treatment for chronic migraine: a prospective, open-label study. Pain Med. (2020) 21:415–22. 10.1093/pm/pnz119 31131857

[21] Starling AJ Tepper SJ Marmura MJ Shamim EA Robbins MS Hindiyeh N. A multicenter, prospective, single arm, open label, observational study of sTMS for migraine prevention (ESPOUSE study). Cephalalgia. (2018) 38:1038–48. 10.1177/0333102418762525 29504483 PMC5944078

[22] Stilling JM Monchi O Amoozegar F Debert CT. Transcranial magnetic and direct current stimulation (TMS/tDCS) for the treatment of headache: a systematic review. Headache. (2019) 59:339–57. 10.1111/head.13479 30671941

[23] Sollmann N Trepte-Freisleder F Albers L Jung NH Mall V Meyer B. Magnetic stimulation of the upper trapezius muscles in patients with migraine—a pilot study. Eur J Paediatr Neurol. (2016) 20:888–97. 10.1016/j.ejpn.2016.07.022 27528122

[24] Renner T Sollmann N Heinen F Albers L Trepte-Freisleder F Klose B. Alleviation of migraine symptoms by application of repetitive peripheral magnetic stimulation to myofascial trigger points of neck and shoulder muscles—a randomized trial. Sci Rep. (2020) 10:5954. 10.1038/s41598-020-62701-9 32249788 PMC7136237

[25] Renner T Sollmann N Trepte-Freisleder F Albers L Mathonia NM Bonfert MV. Repetitive peripheral magnetic stimulation (rPMS) in subjects with migraine-setup presentation and effects on skeletal musculature. Front Neurol. (2019) 10:738. 10.3389/fneur.2019.00738 31379706 PMC6646581

[26] Beaulieu LD Schneider C. Repetitive peripheral magnetic stimulation to reduce pain or improve sensorimotor impairments: a literature review on parameters of application and afferents recruitment. Neurophysiol Clin. (2015) 45:223–37. 10.1016/j.neucli.2015.08.002 26363684

[27] Beaulieu LD Schneider C. Effects of repetitive peripheral magnetic stimulation on normal or impaired motor control. A review. Neurophysiol Clin. (2013) 43:251–60. 10.1016/j.neucli.2013.05.003 24094911

[28] Bartsch T Goadsby PJ. The trigeminocervical complex and migraine: current concepts and synthesis. Curr Pain Headache Rep. (2003) 7:371–6. 10.1007/s11916-003-0036-y 12946290

[29] Bartsch T Goadsby PJ. Central mechanisms of peripheral nerve stimulation in headache disorders. Prog Neurol Surg. (2011) 24:16–26. 10.1159/000323008 21422773

[30] Headache Classification Committee of the International Headache Society (IHS). The international classification of headache disorders, 3rd edition. Cephalalgia. (2018) 38:1–211. 10.1177/0333102417738202 29368949

[31] Fritsche G Hueppe M Kukava M Dzagnidze A Schuerks M Yoon MS. Validation of a German language questionnaire for screening for migraine, tension-type headache, and trigeminal autonomic cephalgias. Headache. (2007) 47:546–51. 10.1111/j.1526-4610.2007.00758.x 17445104

[32] Yoon MS Obermann M Fritsche G Slomke M Dommes P Schilf C. Population-based validation of a German-language self-administered headache questionnaire. Cephalalgia. (2008) 28:605–8. 10.1111/j.1468-2982.2008.01560.x 18422724

[33] Jensen R Tassorelli C Rossi P Allena M Osipova V Steiner T. A basic diagnostic headache diary (BDHD) is well-accepted and useful in the diagnosis of headache. A multicentre European and Latin American study. Cephalalgia. (2011) 31:1549–60. 10.1177/0333102411424212 22019575

[34] Lipton RB Stewart WF Sawyer J Edmeads JG. Clinical utility of an instrument assessing migraine disability: the migraine disability assessment (MIDAS) questionnaire. Headache. (2001) 41:854–61. 10.1046/j.1526-4610.2001.01156.x 11703471

[35] Stewart WF Lipton RB Dowson AJ Sawyer J. Development and testing of the migraine disability assessment (MIDAS) questionnaire to assess headache-related disability. Neurology. (2001) 56(6 Suppl 1):S20–8. 10.1212/WNL.56.suppl_1.S20 11294956

[36] Smania N Corato E Fiaschi A Pietropoli P Aglioti SM Tinazzi M. Therapeutic effects of peripheral repetitive magnetic stimulation on myofascial pain syndrome. Clin Neurophysiol. (2003) 114:350–8. 10.1016/S1388-2457(02)00367-X 12559244

[37] Caminero AB Cuadrado ML Arendt-Nielsen L Pareja JA. Generalized neck-shoulder hyperalgesia in chronic tension-type headache and unilateral migraine assessed by pressure pain sensitivity topographical maps of the trapezius muscle. Cephalalgia. (2010) 30:77–86. 10.1111/j.1468-2982.2009.01901.x 19515127

[38] Baron J Ruiz M Palacios-Cena M Madeleine P Guerrero AL Arendt-Nielsen L. Differences in topographical pressure pain sensitivity maps of the scalp between patients with migraine and healthy controls. Headache. (2017) 57:226–35. 10.1111/head.12984 27885640

[39] Florencio LL Giantomassi MC Carvalho GF Goncalves MC Dach F Fernandez-de-Las-Penas C. Generalized pressure pain hypersensitivity in the cervical muscles in women with migraine. Pain Med. (2015) 16:1629–34. 10.1111/pme.12767 25929269

[40] Grossi DB Chaves TC Gonçalves MC Moreira VC Canonica AC Florencio LL. Pressure pain threshold in the craniocervical muscles of women with episodic and chronic migraine: a controlled study. Arq Neuropsiquiatr. (2011) 69:607–12. 10.1590/S0004-282X2011000500007 21877028

[41] Palacios-Cena M Lima Florencio L Natalia Ferracini G Baron J Guerrero AL Ordas-Bandera C. Women with chronic and episodic migraine exhibit similar widespread pressure pain sensitivity. Pain Med. (2016) 17:2127–33. 10.1093/pm/pnw056 27084411

[42] Akerman S Romero-Reyes M Holland PR. Current and novel insights into the neurophysiology of migraine and its implications for therapeutics. Pharmacol Ther. (2017) 172:151–70. 10.1016/j.pharmthera.2016.12.005 27919795

[43] Dodick DW. A phase-by-phase review of migraine pathophysiology. Headache. (2018) 58 Suppl 1:4–16. 10.1111/head.13300 29697154

[44] Puledda F Messina R Goadsby PJ. An update on migraine: current understanding and future directions. J Neurol. (2017) 264:2031–9. 10.1007/s00415-017-8434-y 28321564 PMC5587613

[45] Blaschek A Decke S Albers L Schroeder AS Lehmann S Straube A. Self-reported neck pain is associated with migraine but not with tension-type headache in adolescents. Cephalalgia. (2014) 34:895–903. 10.1177/0333102414523338 24554618

[46] Landgraf MN von Kries R Heinen F Langhagen T Straube A Albers L. Self-reported neck and shoulder pain in adolescents is associated with episodic and chronic migraine. Cephalalgia. (2016) 36:807–11. 10.1177/0333102415610875 26460336

[47] Sollmann N Mathonia N Weidlich D Bonfert M Schroeder SA Badura KA. Quantitative magnetic resonance imaging of the upper trapezius muscles—assessment of myofascial trigger points in patients with migraine. J Headache Pain. (2019) 20:8. 10.1186/s10194-019-0960-9 30658563 PMC6734472

[48] Sollmann N Schandelmaier P Weidlich D Borner C Urban G Lang M. Patients with episodic migraine show increased T2 values of the trapezius muscles—an investigation by quantitative high-resolution magnetic resonance imaging. Cephalalgia. (2021) 41:934–42. 10.1177/0333102421996374 33615841 PMC8217886

[49] Jensen R. Mechanisms of tension-type headache. Cephalalgia. (2001) 21:786–9. 10.1046/j.0333-1024.2001.00251.x 11595014

[50] Krusz JC. Tension-type headaches: what they are and how to treat them. Prim Care. (2004) 31:293–311. 10.1016/j.pop.2004.02.004 15172508

[51] de Tommaso M Fernandez-de-Las-Penas C. Tension type headache. Curr Rheumatol Rev. (2016) 12:127–39. 10.2174/1573397112666151231113625 26717946

[52] Jay GW Barkin RL. Primary headache disorders- part 2: tension-type headache and medication over use headache. Dis Mon. (2017) 63:342–67. 10.1016/j.disamonth.2017.05.001 28886861

[53] Tassorelli C Diener HC Dodick DW Silberstein SD Lipton RB Ashina M. Guidelines of the international headache society for controlled trials of preventive treatment of chronic migraine in adults. Cephalalgia. (2018) 38:815–32. 10.1177/0333102418758283 29504482

[54] Miller S Watkins L Matharu M. Predictors of response to occipital nerve stimulation in refractory chronic headache. Cephalalgia. (2018) 38:1267–75. 10.1177/0333102417728747 28901169

[55] Barbanti P Ferroni P. Onabotulinum toxin A in the treatment of chronic migraine: patient selection and special considerations. J Pain Res. (2017) 10:2319–29. 10.2147/JPR.S113614 29033605 PMC5628659

[56] Alpuente A Gallardo VJ Torres-Ferrus M Alvarez-Sabin J Pozo-Rosich P. Short and mid-term predictors of response to onabotulinumtoxin A: real-life experience observational study. Headache. (2020) 60:677–85. 10.1111/head.13765 32086801

[57] Quintas S Garcia-Azorin D Heredia P Talavera B Gago-Veiga AB Guerrero AL. Wearing off response to onabotulinumtoxin A in chronic migraine: analysis in a series of 193 patients. Pain Med. (2019) 20:1815–21. 10.1093/pm/pny282 30657951

[58] Schneider C Zangrandi A Sollmann N Bonfert MV Beaulieu LD r PMSCG. Checklist on the quality of the repetitive peripheral magnetic stimulation (rPMS) methods in research: an international delphi study. Front Neurol. (2022) 13:852848. 10.3389/fneur.2022.852848 35392633 PMC8981720

[59] Kisler LB Weissman-Fogel I Coghill RC Sprecher E Yarnitsky D Granovsky Y. Individualization of migraine prevention: a randomized controlled trial of psychophysical-based prediction of duloxetine efficacy. Clin J Pain. (2019) 35:753–65. 10.1097/AJP.0000000000000739 31241488

